# Flexibility of little auks foraging in various oceanographic features in a changing Arctic

**DOI:** 10.1038/s41598-020-65210-x

**Published:** 2020-05-19

**Authors:** Dariusz Jakubas, Katarzyna Wojczulanis-Jakubas, Lech M. Iliszko, Dorota Kidawa, Rafał Boehnke, Katarzyna Błachowiak-Samołyk, Lech Stempniewicz

**Affiliations:** 1 Department of Vertebrate Ecology and Zoology, Faculty of Biology, University of Gdańsk, Wita Stwosza 59, PL-80-308 Gdańsk, Poland; 2 Institute of Oceanology, Polish Academy of Sciences, Powstańców Warszawy 55, PL-81−712 Sopot, Poland

**Keywords:** Ecosystem ecology, Marine biology

## Abstract

Using GPS-tracked individuals, we compared foraging ecology and reproductive output of a High-Arctic zooplanktivorous seabird, the little auk *Alle alle*, between three years differing in environmental conditions (sea surface temperature). Despite contrasting environmental conditions, average foraging fights distance and duration were generally similar in all studied years. Also, in all years foraging locations visited by the little auk parents during short trips (ST, for chick provisioning) were significantly closer to the colony compared to those visited during long trips (LTs, mainly for adults’ self-maintenance). Nevertheless, we also found some differences in the little auk foraging behaviour: duration of LTs was the longest in the coldest year suggesting more time for resting for adults compared to warmer years. Besides, birds foraged closer to the colony and in significantly colder water in the coldest year. Interestingly, these differences did not affect chick diet: in all the years, the energy content of food loads was similar, with the Arctic copepod, *Calanus glacialis* copepodite stage V being the most preferred prey item (>73% of items by number and >67% by energy content). Also chick survival was similar in all the study years. However, when examining chicks growth rate we found that their peak body mass was lower in warmer years suggesting that overall conditions in the two warm years were less favourable. While our results, demonstrate a great foraging flexibility by little auks, they also point out their vulnerability to changing environmental conditions.

## Introduction

Optimal foraging theory predicts that animals should adjust their foraging behaviour to environmental condition the way to maximise net energy gain and to increase their survival and/or reproductive success^1^. The ability to efficiently adjust foraging behaviour should be especially apparent in central place foragers, such as seabirds that forage at distant locations from which they need to regularly return to the colony to feed their young^2^. Prey distribution and abundance vary spatially and temporally in response to macroscale fluctuations of oceanographic conditions (e.g., North Atlantic Oscillation, El Niño Southern Oscillation;^3,4^ as well as to mesoscale processes [e.g., sea ice dynamics, sea current distribution, upwelling strength and spread, atmospheric blocking; e.g.^5–9^]. Seabirds are generally well adapted to the foraging in such the heterogeneous marine habitat. However, the recent climate change increases additional, often unpredictable fluctuations in prey abundance^10,11^. It is specially visible in the Arctic where both marine and terrestrial ecosystems are rapidly changing as climate warming affects these regions faster than the global average^12–15^. Moreover, high-latitude nesting seabirds have a limited time window to reproduce, which may limit their ability to adjust the timing of breeding to shifts in peaks of food abundance^7^. Thus, Arctic endemic species are extremely susceptible to climate change. In the high Arctic marine ecosystem, increase in sea temperature and reductions in sea ice are influencing the entire food web. These changes are affecting the foraging and breeding ecology of many marine birds and mammals^12,13^.

Here, we study foraging ecology of an endemic Arctic avian predator, the little auk (or dovekie *Alle alle*). It is a small zooplanktivorous seabird breeding colonially, exclusively in the High Arctic^16^. Its diet during the chick-rearing period in Svalbard consists mainly of Arctic copepods, that are larger and much richer in energy than their counterparts from warmer Atlantic waters^17–19^. Little auks lay a single egg which is incubated by both parents. The chick is brooded for the first few days and fed by both parents^16^. Due to its abundance (37–40 million pairs^20,21^), the little auk is an important component of High Arctic ecosystems and transports huge amounts of organic matter from sea to land, fertilizing the nutrient-deprived Arctic tundra^22,23^. Considering its number, diet, and breeding range, this seabird is an excellent model species to study response of endemic Arctic organism to currently observed climate change. Previous studies revealed that despite apparent flexibility of foraging little auks, suboptimal environmental conditions affect negatively chick growth and annual survival rate^24–28^. However, these investigations have not addressed consequences of the inter-annual variability in environmental conditions and energy density of food for the little auk foraging flights characteristic and breeding performance.

We aimed to compare foraging flights of GPS-tracked little auks breeding in Hornsund (SW Spitsbergen) between three years characterized by various environmental conditions. We took advantage of inter-annual variability of the sea surface temperature (SST) and volume of Atlantic waters along the west coast of Spitsbergen including the Hornsund area^29^ and compared available GPS-tracking data from a cold summer 2011 characterized by lower SST caused by the sea ice inflow brought by the Sørkapp Current from the Barents Sea^30^ and two other warmer years (2016 and 2018) without sea-ice close to the colony. We also investigated how foraging in various environmental conditions affect chick diet composition, and breeding performance (chick survival and chick growth parameters).

Animals may maximize fitness optimizing their energy budget (by maximizing energy intake and minimizing energy expenditure) through the selection of preferred foraging habitats^31–33^. Thus, we expected that in the coldest year little auks would forage in nearby Arctic waters and the marginal ice zone (MIZ), optimal habitats for their preferred zooplankton prey, while in the warmer years they would try to find alternative foraging grounds rich in optimal prey (e.g. in the Polar Front, i.e. thermal front separating colder Arctic and warmer Atlantic water masses located over the shelf break^34,35^). However, little auks may face various limitations when foraging areas are located further from the colony and/or characterized by food of worse quality or/and quantity^36^. We therefore expected that despite documented little auk foraging flexibility^37–40^, strength of ‘Atlantification’ in the warmer years would result in increased parental expenditures reflected in longer flights to more distant locations and/or spending more time on foraging in suboptimal conditions. We also expected that increased parental expenditures (longer flights) in warmer years would result in worse reproductive performance (in terms of breeding success and chick body mass) compared to the cold year.

The optimal foraging theory also assumes that if various prey taxa are abundant in the environment, predators display preference and specialize on a superior prey type that guarantees the best net energy gain^41^. Thus, we predicted that foraging little auks would deliver mostly large energy-rich zooplankton prey (i.e. cold-water copepods and sympagic amphipods) for their chicks.

During the chick-rearing period little auks adopt a dual foraging, alternating long trips (LT) with several consecutive short trips (ST) lasting in SW Spitsbergen for 1.3–2.0 h and 11.7–13.0 h, respectively^25,42–45^. The study of body mass of adults returning to nest burrows from the foraging flight revealed that during STs birds collect food for chicks while LTs are mainly dedicated to self-maintenance^46^. As the duration of LTs may be affected by environmental conditions [in contrast to STs, the duration of LT increased with increasing SST^42^], we investigated whether year or trip type (ST or LT) affect flight parameters (duration, maximal ranges, and total distance covered), and whether LTs and STs are directed to areas differing in environmental conditions (SST, depth and distance from the thermal front). We expected longer and further LTs in warmer years and STs of similar range and duration in all studied years. Considering the shorter duration of STs, we expected that birds during this type of trips would visit closer locations compared to further locations during LTs.

## Methods

### Study area

The study was performed in the breeding colony in Hornsund (SW Spitsbergen), considered as one of the largest little auk breeding aggregation in Svalbard^21,47^. The Hornsund area is influenced by both the coastal, Sørkapp Current transporting cold Arctic masses and the warm West Spitsbergen Current (WSC) that transports warmer Atlantic water masses from the Norwegian Sea^48^. These two distinct external water masses are usually separated outside the fjord by a hydrological front (Arctic or Polar Front)^34,35^ but the range and distribution of the Atlantic and Arctic water masses vary considerably among the years. Sea ice in the area is present only in some years depending on atmospheric and oceanic processes^24,39^. Due to a considerable contribution of cold Arctic water masses, foraging grounds in the Hornsund area are considered as favourable for Arctic zooplankton and consequently for the little auk^37–39,49^. However, it has been documented that environmental conditions vary between the years affecting energy value of little auk chicks diet^24,39^.

### GPS-tracking

To recognized birds foraging area and study characteristics of foraging flight, we used miniature GPS loggers (Ecotone, Sopot, Poland) of two types: EP (size 40 × 17 × 9 mm) in 2011 and ALLE (size 27 × 16 × 12 mm) in 2016 and 2018. The GPS logger was attached to the bird’s central back feathers using four transversally applied, 2 mm wide strips of Tesa tape (code 4965, Tesa Tape Inc. Charlotte, NC, USA) at approximately the midpoint of the centre-line of the body. The logger weight (4.2–4.5 g including attachment) was equivalent to 2.3–3.3% of the little auk’s body mass^38,44,50^ and was concordant with generally accepted recommendation that the weight of the device should not exceed ca 5% of a bird’s body mass^51^. The GPS-loggers used bidirectional radio link with base stations installed in the colony, allowing remote data download, without necessity of bird recapture.

In total, we deployed loggers on 40 individuals in the three study years (Supplementary Materials, Table S1). Generally, we deployed the loggers on one pair member only, although in 2016 and 2018 we also deployed loggers on both pair members (sequentially not simultaneously) in 3 and 1 nest, respectively. Of all the deployed loggers, we obtained records from 37 individuals (92.5% instrumented), originating from 33 nests (Table S1). Data series from 10 deployed individuals (27% of all with collected data) were too short for analysis (they contained only data from the colony; birds may have lost loggers soon after deployment or did not return to the colony) and we excluded them from further analysis. Thus, in total we analysed 81 GPS-tracks (86.2% of all recorded) of 28 birds (70% of all individuals with deployed loggers, and 75.7% of individuals with recorded data) (Table S1).

**Figure 1:**
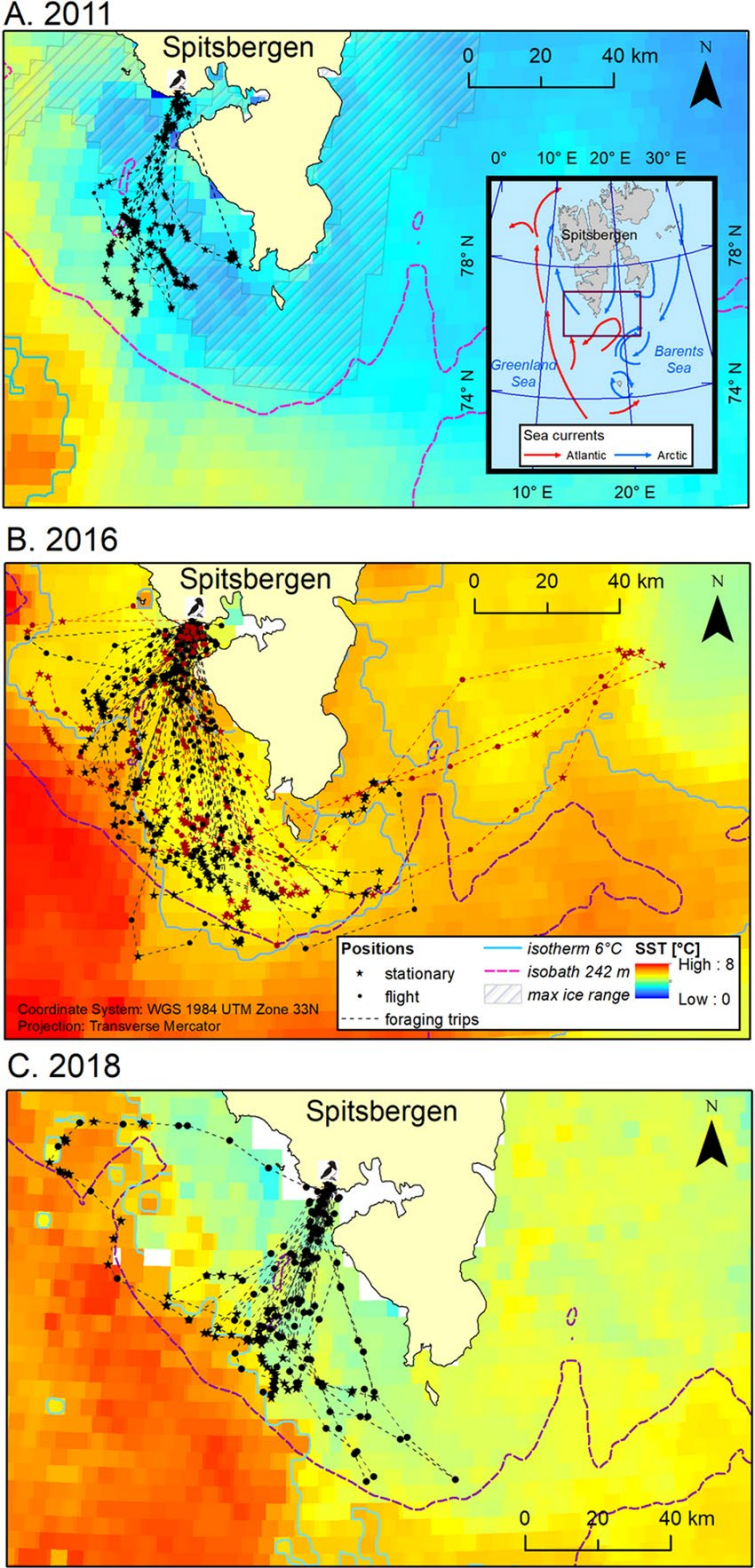
Foraging trips of chick-rearing GPS-equipped little auks breeding in Hornsund in 2011 (**A**) 2016 (**B**), and 2018 (**C**). Sea surface temperature (SST) maps – monthly mosaic for July based on MODIS Aqua satellite data^53^]; mosaic for July 2016 reconstructed based on mosaics for July and August 2016 (see details in the text). max ice range – maximal range of sea ice during the period of recorded trips of GPS-equipped little auks in 2011; source: Multisensor Analyzed Sea Ice Extent - Northern Hemisphere (MASIE-NH), Version 1^55^. A 242 m isobath reflects the multiyear cut-off point value for Arctic zooplankton occurrence in the Hornsund area^39^; isobath created based on global relief model of Earth’s surface IBCAO ver. 3^54^. Isotherm 6 °C (for the studied period in particular years) reflects physiological threshold for *Calanus glacialis* functioning in Svalbard^67^. Zoom out in A panel – study area with the frame indicating area shown in the zoom in maps. Arrows indicate ocean currents [after^35^, modified]. In 2016, black and brown lines and points indicate flights and GPS positions of successful and failed breeders, respectively. Maps were produced in ArcMap 10.3.1 (Redlands, CA: Environmental Systems Research Institute).

Loggers deployment did not have apparent negative effect on birds’ survival: in most of the nests with GPS-deployed adult/s the chick was found alive during the two consecutive control visits after the logger deployment (N = 32 nests; 88.9% of nests with individuals deployed with the logger). Only in three nests (8.3%) the chick was found dead during the control nest visit. For this reason, we excluded data from dead chick parents from main analyses and showed their trips separately in Fig. 1.

Data from GPS tracked individuals in 2011 have been already published^38^ but here we present them in comparison with analogous data from the two other years, 2016 and 2018. Data from 2016 and 2018 have not been published anywhere yet, and are presented here for the first time.

All animal research protocols were carried out in accordance with guidelines for the use of animal^52^ and approved by Norwegian Animal Research Authority and the Governor of Svalbard. More details about protocols and effects of loggers on birds are provided in Supplementary Materials (Effect of GPS loggers on the chicks growth rate) and previous studies^38,50^.

### Environmental conditions

To characterize environmental conditions at foraging grounds, we used remote-sensed satellite data on sea depth, sea surface temperature (SST), and sea ice extent. Those features have been recognized as most important determinants of the occurrence of Arctic zooplankton in Svalbard^39,49^, thus also in little auk foraging areas. We extracted SST for the foraging locations (see below) of little auks from the Moderate-resolution Imaging Spectroradiometer (MODIS) Aqua satellite data. We used Level 3 daytime SST data derived from 11 μm thermal IR infrared (IR) bands with a 4 km spatial resolution^53^. For 2011 and 2016 we used SST monthly mosaics for July that represents the early and mid chick-rearing periods, when the GPS-tracking data were collected (Table 1). In 2018, due to high cloudiness during the whole July, SST maps for this month were characterized but many areas with no available data. To characterize thermal conditions in this year we were forced to reconstruct SST values based on monthly mosaics for two months, July and August. To do so, we firstly created isopleths of 95% utilization densities for foraging locations of breeders in 2018 (see details in Data analyses). Within mentioned 95% utilization densities we generated randomly 500 points. Then we extracted SST values for those points from monthly mosaics for July and August. We were able to extract data for 252 points with available values of SST in both monthly mosaics. As the values for July (mean ± SD: 5.9 ± 1.55 °C) and August (6.6 ± 0.93 °C) differed significantly (paired Student *t* test: t = 8.45, p < 0.0001), we created the monthly mosaic for July with reconstructed SST values by subtracting mean difference between the months (0.7299 °C) from the August mosaic.

**Table 1:** Characteristics of recorded foraging trips of chick-rearing GPS-logger equipped little auks breeding in Hornsund in 2011, 2016 and 2018.

Year	Bird ID	Maximal [km]…		No.	Chick age [d]		No of..	
		flights range	distance cover	fixes	Min	Max	LTs	STs
2011	H11	51	123	58	2	2	1	—
	H12	54	143	66	7	7	1	—
	H19	61	164	89	2	3	2	—
	H5	61	146	43	2	2	1	—
	HS05	5	13	18	1	1	—	2
	Total/Max	61	164	274	1	7	5	2
2016	ALL01	51	105	23	2	5	3	—
	ALL02	22	46	27	3	4	2	2
	ALL03	51	135	18	3	5	1	2
	ALL04	36	77	34	4	6	2	1
	ALL05	23	64	18	2	4	1	2
	ALL08	2	3	3	4	4	—	1
	ALL09	79	185	165	5	11	8	6
	ALL10	88	213	34	3	6	4	1
	ALL11	40	80	5	4	4	1	—
	ALL12	89	219	83	4	8	3	4
	ALL23	100	326	94	5	10	4	3
	Total/Max	100	326	504	2	11	29	22
2018	ALL07	7	14	9	1	2	—	2
	ALL08	38	77	15	5	5	—	3
	ALL04A	8	15	3	5	5	—	1
	OLD01A	87	211	90	4	6	4	—
	OLD02	64	164	60	4	8	3	2
	ALL09B	59	177	31	8	9	1	2
	OLD01B	57	130	45	12	14	1	2
	ALL06	35	70	4	5	5	—	1
	ALL04B	34	69	3	9	9	—	1
	Total/Max	87	211	260	1	14	9	14
All	Total	100	326	1038	1	14	43	38

No. fixes – total number of GPS fixes recorded for particular individuals, LTs – long trips, STs – short trips. Chick age – min and max age of chicks when the one of parents was GPS-tracked. Total/Max – total number of LTs and STs and all GPS fixes, and maximal values of maximal range of flights and oral distance covered and maximal age of chick life in particular year.

We extracted sea depth data for the foraging positions from a 500 m global relief model of Earth’s surface IBCAO ver. 3^54^. We visualized sea ice extent using daily maps of sea ice extent from Multisensor Analyzed Sea Ice Extent - Northern Hemisphere (MASIE-NH), Version 1 with 4 km grid cell size^55^.

We extracted all abiotic data from GIS data and created the monthly mosaic for July 2018 with reconstructed SST values using ArcGIS software 10.3.1 (Redlands, CA, USA: Environmental Systems Research Institute).

### Little auk chick diet composition, growth and survival

To test the effect of the environmental condition on the birds’ diet (composition and energy content) we collected food samples from adults captured in the colony, when transporting food to their chicks. We captured the birds using noose-carpets or/and lines with loops. After the capturing, we gently scooped out food content from the bird’s gular pouch (special sublingual sac to transport food for chick) with a small spoon. We put each food load in a separate plastic container and preserved in 4% formaldehyde solution. In total, we collected 27 diet samples in 2011, 20 in 2016, and 20 in 2018 at the mid phase of chick-rearing (at the age of chicks of 2 weeks). We released the sampled birds after 5–10 min of handling. We analysed collected food samples quantitatively and qualitatively following the procedures described in^56^, and identifying copepods from the genus *Calanus* to species and developmental stage in accordance with^57^.

To examine the reproductive output of the little auk in the studied breeding seasons we monitored chick growth parameters and survival, based on control nests (i.e. with parents not burdened with GPS-loggers). For chick growth rate, we monitored body mass of chicks in the group of 66, 18, 23 control nests with known date of hatching in 2011, 2016 and 2018, respectively. We started body mass controls when the chicks were at age 14 to 15 d. We weighed them every 3 d until they disappeared from the nest (at 21 to 31 d^58^). Of that we calculated peak body mass (the highest mass noted per chick), fledging mass (the last mass measured before the chick’s departure from the colony), both considered as effective growth indicators, even more than the widely used growth-curve analysis^59–61^. We also established and used in further analysis the day when peak mass occurred and the day of fledging (the last presence in the nest). As we were not able to catch few chicks during all the visits, the sample sizes differ for particular body mass parameters. For the same reason, we analysed growth rate parameters only for chicks weighed at least 3 times. In total, we followed until the fledging: 64 chicks in 2011, 11 in 2016 and 23 in 2018. We also followed additional 2 chicks in 2011, 7 in 2016 and 5 in 2018 until age of 20 d (we were not able to catch them during the last control before fledging); we were not able to analyse their fledging mass but we did it for the peak mass.

### Data analyses

Based on the geographical positions recorded by the GPS-loggers, for each of the foraging flight we established: (1) maximum range of flight – distance (km) from the colony to the distal point reached during the trip, (2) the total distance covered (km) as the sum of the distances (km) between all GPS positions along the track, and (3) total trip duration (h), defined as the time between departure and return to colony.

We distinguished foraging locations based on momentary flight speed (km h^−1^) recorded instantaneously by GPS-loggers. For that, we assumed values ≤10 km h^−1^ as foraging location as they are considered to be associated with swimming and feeding^62,63^. Sampling interval was set to 15 min, and in practice we obtained records characterized by 15–20 min intervals (logger was trying to reach the GPS signal after failure at scheduled time, when e.g. birds was diving). The field-tested accuracy of the GPS receiver was ±10 m for 95% of positions.

Due to evident bimodal distribution of foraging flights duration, we divided foraging trips (from all years combined) into long (LTs) and short trips (STs) using a cut-off value of 8 h for total trip duration, calculated as the minimal sum of the variances of both trip types given their non-normal distribution [see details in^42^].

To examine significance of the year as a determinant of flight distance and duration we applied modelling approach (linear mixed models, LMM). For that we considered three separate models with maximum range of flights, total distance covered, or total trip duration as response variables. We performed the three separate models because when analysing the maximum range of flights or total distance covered as the response variable we considered the issue from the perspective of adults’ energy expenditures, while in the model with foraging trip duration, adults’ time budget was considered. In all the models, apart from the year being the explanatory variable (factorial predictor) we also included trip type (LT, ST, factorial), and chick age (continuous). For the models with maximum range and total distance covered we additionally included trip duration as a continuous predictor, and for the model with total trip duration, the total distance covered as the continuous predictor. Since in all the models multiple tracks of the same individuals were considered we also included bird identity as a random factor to account for possible pseudoreplication issue. Since the effect of trip type was significant in all the models (see Results), we performed all these analyses, separately for LTs and STs, with an obvious difference – exclusion of the trip type from the predictors.

To analyse environmental conditions at the true foraging areas (i.e. at the areas utilized by birds in a given season as revealed by the GPS-tracks) in the three years we compared SST values in randomly generated points within the 95% utilization densities of all foraging locations between the studied years. For that purpose, we firstly extracted SST from annual July mosaics values for 500 random points. Due to lack of data in some areas (due to high cloudiness in some areas in some years) we obtained SST values from all three years for 163 points. Then, we compared the SST values between years using Friedman test for equality of medians in repeated-measures groups with pairwise Wilcoxon tests as *post-hoc* tests.

To further analyse environmental conditions at the foraging grounds, we also detected thermal (SST) fronts using Cayula & Cornillon algorithm^64,65^ with a 1 °C threshold in SST MODIS Aqua satellite images mentioned before in Marine Geospatial Ecology Tool toolbox^66^ in ArcGIS software 10.3.1. Then we calculated distance from foraging position to thermal fronts (the Euclidean distance from the foraging locations to the front).

To characterize various microhabitats by foraging little auks we distinguished four distinct thermal zones:

1. marginal ice zone (MIZ) with SST ranging from minimal recorded values to 2.7 °C;
2. cold water zone with SST from 2.71 °C to 5.1 °C [SST < 5.1 °C has been recognized as a range optimal for the Arctic zooplankton community occurring in the Hornsund area^39^];
3. suboptimal zone 5.11 °C – 6.0 °C [isotherm 6 °C reflects physiological upper threshold for *Calanus glacialis* functioning in Svalbard^67^];
4. warm water zone with SST > 6.0 °C.

Then we calculated proportions of foraging locations in particular thermal zones between the studied years. We also compared proportion of foraging locations in frontal zone and outside (with an arbitrary cut-off value of 2.5 km for the distance from the nearest SST front). We compared proportion of foraging locations in particular zones using chi-squared (thermal zones) or Fisher exact (frontal vs non-frontal zones) tests.

We also analysed little auk foraging areas applying a Conditional Inference Tree (CIT). This is a non-parametric class of regression tree, examining the relationship between multiple explanatory variables and a single response variable using a recursive binary-partitioning process. Model outputs produce an ‘inverted tree’, in which the root at the top contains all observations, which is divided into two branches at the node. The aim of splitting the data at each step is to establish groups that had a between-variation as large, and within-variations as small, as possible. The node provides information about the explanatory variable name and its probability value. Branches are further split into two subsequent nodes and so on^68^. CIT uses a machine learning algorithm to determine when splitting is no longer valid using statistically-determined stopping criterion, an a priori p value^69^. CIT is robust to typical regression problems such as over-fitting, collinearity, and bias with regard to the types of explanatory variables used^69^. Thus, we performed CIT analyses with sea surface temperature, sea depth, distance from the colony or distance to the thermal front as a response variable (all the variables in separate analyses) and year and foraging trip type (LT or ST) as factorial predictors.

To examine the chick diet we considered abundance and energy content of the abundant prey items, i.e. those constituting ≥1.5% of total energy content. We compared these parameters among the study years using Kruskal-Wallis test. We calculated total energy value of food samples based on the particular prey energy estimates according to^19,70^. To compare total energy value of food loads between the years we used Kruskal-Wallis test.

To compare breeding success among particular years we chose chick survival up to 20 days [i.e. the number of 20-day-old chicks / number of chicks hatched; chicks that disappeared from the nest after 20 days were assumed to have fledged;^71^] using Fisher’s exact test with p–values calculated by Monte Carlo simulation.

To examine the effect of the year (factorial predictor) on the chick growth rate in control nests, we performed 1) linear models (LM) with peak body mass or fledging body mass as a response variable and 2) generalized linear models with Poisson distribution (GLM) with body mass day or fledging day as the response variable. In all the modes we included additional covariates: chick age at the time of achieving the peak mass or fledgling for the two models considering chick body mass, and peak or fledging body mass in two other models considering chicks age at the moment of achieving body mass or fledging.

For all linear analyses we assessed whether the data sufficiently met relevant assumptions using Q–Q plots (quantile expected in normal distribution vs quantile observed plot for residuals) and Shapiro-Wilk test. We also checked multicollinearity using variation inflation factor and accepted only models with VIF < 5^72^. When the assumptions were violated, we either performed log, 1/x or Box-Cox transformation data or used non-parametric test.

In all LMM and LM analyses we used *post-hoc* estimated marginal means test or Tukey test with Holm correction. To estimate significance of the random effect in LMM analyses we compared models with and without random effect using *F*-test with Kenward-Roger approximation^73^. We performed LMM analyses in *lmer*, *lme4*, *pbkrtest* packages^73,74^, *post-hoc* estimated marginal means test in *emmeans* package, *post-hoc* Tukey test in *multcomp*^75^ variation inflation factor calculated in *car* package, and performed CIT analyses in *partykit* package^76^ in R software^77^. We mapped data from the GPS loggers, extract data from maps and produced all figures with maps using ArcMap/ArcGIS 10.3.1 (Environmental Systems Research Institute, Redlands, CA, USA).

To visualise foraging areas exploited by birds we calculated utilization distributions (UD, a probability distribution constructed from data providing the location of an individual in space at different points in time) for main feeding areas (95% kernel density) and core feeding areas (50% kernel density) in Geospatial Modelling Environment (GME) ver. 0.7.4.0 software (www.spatialecology.com/gme/) using *kde* function using plugin bandwidth selection. We did it for all foraging positions in particular years and then for foraging positions recorded during LTs and STs in particular years. For both the main and core foraging areas, we also calculated the overlap among the years, and between LTs and STs (separately for each year). For that we used *adehabitatHR* package^78^ in R^77^ with algorithm BA, i.e. the Bhattacharyya’s affinity, a statistical measure of affinity between two populations. This measure ranges from zero (no overlap) to 1 (identical UDs)^78,79^.

## Results

### Environmental conditions in the studied years

Sea water temperatures (SST) in the same 163 random points located in the little auk foraging areas differed significantly between the studied years (Friedman test, χ^2^ = 253.6, df = 2, p < 0.001). SST values in 2011 (min - max: 2.2–4.9 °C, median: 3.0 °C, IQR: 0.86) were significantly lower compared to 2016 (min - max: 5.1–7.6 °C, median 5.9 °C, IQR 0.64, Wilcoxon, p < 0.001) and 2018 (min - max: 2.4–8.7 °C, median 6.3 °C, IQR 1.67, Wilcoxon, p < 0.001). Values recorded in 2016 were significantly higher compared to 2018 (p < 0.001).

### General characteristics of foraging flights and foraging locations

**Figure 2:**
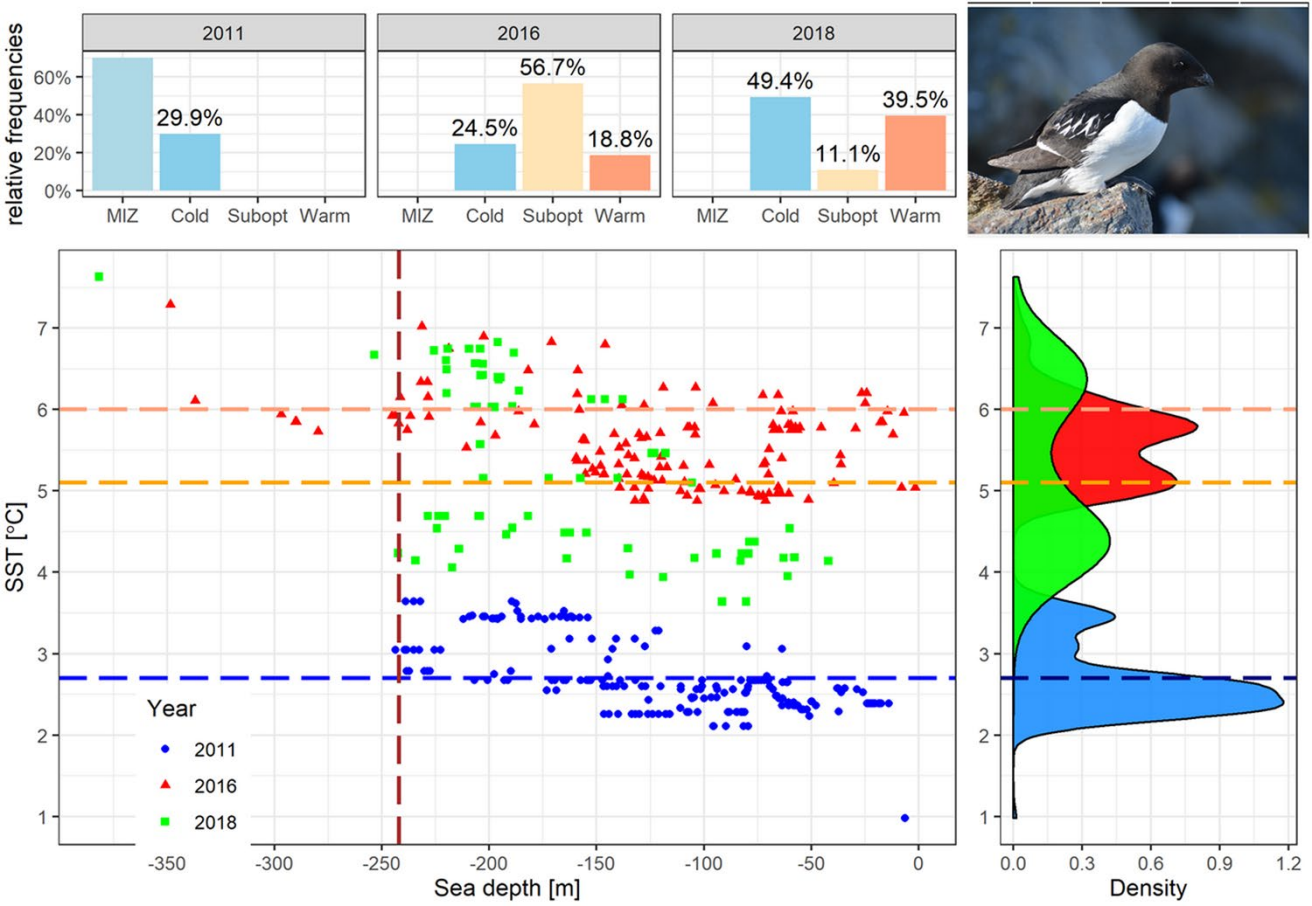
Distribution of sea surface temperature (SST) and sea depth at foraging positions of GPS-logger-equipped chick-rearing little auks breeding in Hornsund in 2011, 2016 and 2018. Density – probability density. Top left graph shows relative frequency of foraging locations in four thermal zones in particular years. MIZ - marginal ice zone with SST ranging from minimal recorded values to 2.7 °C, Cold - cold water zone with SST from 2.71 °C to 5.1 °C [SST < 5.1 °C has been recognized as a range optimal for the Arctic zooplankton community occurring in the Hornsund area^39^]; Subopt - suboptimal zone 5.11 °C – 6.0 °C [isotherm 6 °C reflects physiological upper threshold for *Calanus glacialis* functioning in Svalbard^67^], Warm - warm water zone with SST > 6.0 °C. The bottom left graph shows SST and sea depth of all foraging locations in particular years. Horizontal dashed lines indicate: blue – upper boundary of marginal sea ice zone, orange - the upper threshold of the Arctic zooplankton community occurring in the Hornsund area^39^, red - a physiological upper threshold for *Calanus glacialis* functioning in Svalbard^67^. Red vertical dashed line indicates a 242 m isobath reflecting the multiyear cut-off point value for Arctic zooplankton occurrence in the Hornsund area^39^. Right bottom graph shows distribution of SST in particular years. Density – probability density. Photo of adult little auk in the breeding plumage taken in the breeding colony at Hornsund by Katarzyna Wojczulanis-Jakubas. Plot was created in R software version 3.5.2^77^.

Overall, during the study period, foraging flights (n = 81) performed by little auks lasted from 0.4 to 44.1 h (median 10.8 h, IQR = 11.9), covered distances from 3.0 to 326.0 km (median: 79.5 km, IQR = 122.0), with maximal ranges from 1.5 to 99.6 km (median 39.9 km, IQR = 50.8) per trip. Birds were foraging at locations of 1.6–93.2 km from the colony (median 46.5 km, IQR = 21.6, n = 494 locations), with SST at foraging positions ranged from 1.0 to 7.6 °C (median 3.5 °C, IQR = 2.8). Foraging positions were located at different frontal and thermal zones, although marginal ice zone (with SST ≤ 2.7 °C) was exploited only in 2011; majority of recorded positions in 2011 were located in this marginal ice zone (70.1%; Fig. 2). Foraging locations in cold water zone (2.7 °C > SST ≤ 5.1 °C) were visited by little auks in all studied years and constituted from 24% to 46% of all visited foraging locations. Foraging locations in warmer thermal zones (SST > 6 °C) were recorded exclusively in 2016 and 2018 (Figs. 1 and 2). Foraging locations with seawater temperatures between 5.1 °C > SST < 6.0 °C (i.e. suboptimal for the preferred prey, *C. glacialis* in the Hornsund area) dominated in 2016 making up 57% of foraging positions. In 2018 birds foraged mainly in two thermal zones – in cold water (49%) and warm water zone (SST > 6 °C, unfavourable for Arctic zooplankton) (39%) (Fig. 2). These proportion of locations within cold, suboptimal and warm water zones differed significantly between the studied years (χ^2^ test, χ^2^ = 57.9, df = 2, p = 0.001).

The distribution of sea depth at the foraging locations (min-max: −1.6–382.0 m, median = −126.0 m, IQR = 103.0) overlapped in all studied years. Foraging locations with depth values < −242 m (i.e. suboptimal for the Arctic zooplankton community in the Hornsund area) made up 0.8% in 2011, 7.2% in 2016 and 3.7% in 2018. The sea ice was present on the foraging grounds only in 2011 (Figs. 1 and 2).

We recorded foraging locations situated in the thermal front zone (<2.5 km from the front) only in 2016 and 2018. They made up 8.6% and 11.1% of all stationary positions in 2016 and 2018 respectively. Their proportions in both years were similar (Fisher exact test, p = 0.64).

Two parental birds burdened sequentially with loggers after chick loss performed long-lasting and long-distance trips. Two of three trips of one of the individuals were directed to the furthest foraging areas (with the furthest locations 123 and 129 km from the colony, during one and the second trip, respectively) with relatively cold water (Fig. 1).

### Factors affecting all foraging trips characteristics

**Figure 3:**
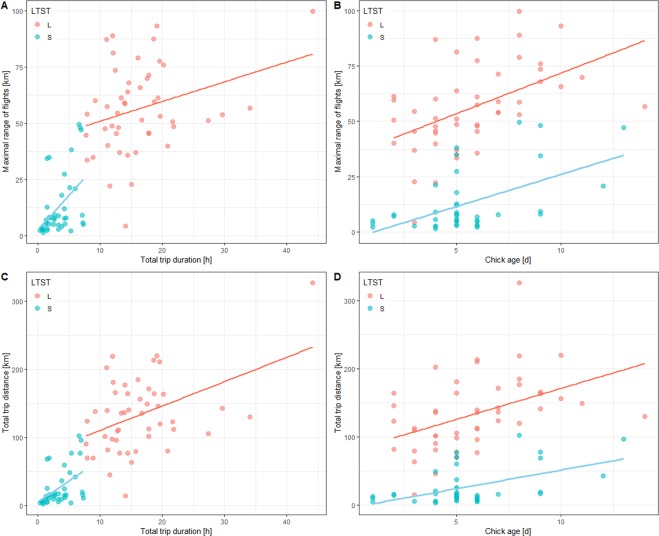
Relationship between distances of foraging flights [maximum range of flights (**A,B**) or total distance covered (**C,D**)] and total trip duration (**A,C**) of GPS-logger equipped little auks from the Hornsund colony and age of their chicks (**B,D**) in 2011, 2016 and 2018, combined. Least squares regression lines are shown for significant relationships. Red points and lines indicates short flights, blue points and lines long flights. Plot was created in R software version 3.5.2^77^.

The maximal range of flights was affected significantly by trip type. The maximal range of LTs (mean ± SE: 58.1 ± 3.0 km, min-max: 4.4–99.6 km, N = 43) was significantly higher compared to STs (mean ± SE: 16.2 ± 2.7 km, min-max: 1.5–53.9 km, N = 38). The maximal range of flights increased significantly with chick age (LMM, estimate ± SE: 2.88 ± 0.84) and increasing total flight duration (estimate ± SE: 0.82 ± 0.33) (Fig. 3A,B). It was also affected significantly by a random effect (bird identity). Effect of year was not significant (Table 2).

**Table 2:** Linear mixed effects models estimating the effects of year (factorial: 2011, 2016, 2018), foraging trip type (factorial: long vs short trips), chick age (continuous) and foraging flight duration/total trip distance (continuous), and bird identity (random effect) on characteristics of foraging flights of GPS-tracked little auks.

All trips	Maximal range				Total distance covered					log Total flight duration			
	ndf	ddf	F	P	ndf	ddf	F	P	All trips	ndf	ddf	F	P
Year	2	25.8	2.5	0.098	2	26.5	2.0	0.159	Year	2	12.0	1.6	0.246
Trip type	1	68.1	31.1	**0.000**	1	67.3	16.9	**0.000**	Trip type	1	74.9	35.8	**0.000**
Chick Age	1	54.3	11.7	**0.001**	1	57.3	6.0	**0.017**	Chick Age	1	24.0	0.1	0.755
Total Durat.	1	66.2	5.9	**0.017**	1	65.6	18.9	**0.000**	Total Dist.	1	71.4	8.7	**0.004**
r.e. bird ID	5	46.4	1.0	**0.000**	5	47.0	1.0	**0.000**	r.e. bird ID	5	40.9	1.0	**0.000**
**Short trips**	**log Maximal range**				**log Total dist. covered**					**log Total flight duration**			
	**ndf**	**ddf**	**F**	**P**	**ndf**	**ddf**	**F**	**P**	**Short trips**	**ndf**	**ddf**	**F**	**P**
Year	2	12.2	1.8	0.215	2	12.5	1.4	0.273	Year	2	4.1	0.11	0.903
Chick Age	1	21.4	6.5	**0.018**	1	21.5	6.2	**0.021**	Chick Age	1	11.9	1.39	0.262
Total Durat.	1	32.7	17.6	**0.000**	1	32.8	20.4	**0.000**	Total Dist.	1	30.8	17.4	**0.000**
r.e. bird ID	4	18.7	1.0	**0.000**	4	18.7	1.0	**0.000**	r.e. bird ID	4	17.8	0.99	**0.007**
**Long trips**	**Box-Cox Max. range**				**Total distance covered**					**1/Total flight duration**			
	**ndf**	**ddf**	**F**	**P**	**ndf**	**ddf**	**F**	**P**	**Long trips**	**ndf**	**ddf**	**F**	**P**
Year	2	16.4	0.5	0.634	2	15.98	0.26	0.771	Year	2	12.94	5.49	**0.019**
Chick Age	1	37.5	8.9	**0.005**	1	38.00	1.95	0.170	Chick Age	1	27.32	7.55	**0.010**
Total Durat.	1	28.2	1.5	0.237	1	29.35	9.23	**0.005**	Total Dist.	1	33.34	1.85	0.183
r.e. bird ID	4	25.5	1.0	**0.010**	4	25.07	0.98	**0.005**	r.e. bird ID	4	19.25	0.99	**0.007**

Significance of random effect, bird identity (r.e. bird ID) estimated by *F*-test with Kenward-Roger approximation. Significant effects are bolded.

The total distance covered by birds during the trip was affected significantly by trip type. The total distance covered during LTs (mean ± SE: 137.0 ± 8.6 km, min-max: 14.7–326.0 km, N = 43) was significantly higher compared to STs (mean ± SE: 33.3 ± 5.4 km, min-max: 3.0–124.0 km, N = 38). Other parameters included in the model are reported in Table 2 but considered in detail below, when performing the models separately for the trip type.

The total trip duration (log-transformed) was also affected significantly by trip type. The total duration of LTs (mean ± SE: 16.8 ± 1.0 h, min-max: 8.9–44.1 h, N = 43) was significantly higher compared to STs (mean ± SE: 3.8 ± 0.4 h, min-max: 0.4–7.9 h, N = 38).

Other parameters included in the model are reported in Table 2 but considered in detail below, when performing the models separately for the trip type.

### Factors affecting ST characteristics

The maximal range of STs (log-transformed) increased significantly with chick age (LMM, estimate ± SE: 0.14 ± 0.05) and increasing total flight duration (estimate ± SE: 0.22 ± 0.05) (Table 2, Fig. 3). It was also affected significantly by a random effect (bird identity). Effect of year was not significant (Table 2).

The total distance covered by birds during STs (log-transformed) increased significantly with chick age (LMM, estimate ± SE: 0.13 ± 0.05) and with increasing total flight duration (estimate ± SE: 0.23 ± 0.05) (Table 2, Fig. 3). It was also affected significantly by a random effect (bird identity). Effect of year was not significant (Table 2).

The total trip duration of STs increased significantly with increasing total distance covered (LMM, estimate ± SE: 0.05 ± 0.01). It was also affected significantly by a random effect (bird identity). Other effects (year, chick age) were not significant (Table 2).

### Factors affecting LT characteristics

The maximal range of LTs (Box-Cox-transformed) increased significantly with chick age (LMM, estimate ± SE: 4.84 ± 1.63) (Table 2, Fig. 3). It was also affected significantly by a random effect (bird identity). Other effects (year, total flight duration) were not significant (Table 2).

The total distance covered by birds during LTs increased significantly with increasing total flight duration (estimate ± SE: 3.35 ± 1.10) (Table 2, Fig. 3). It was also affected significantly by a random effect (bird identity). Other effects (year, chick age) were not significant (Table 2).

The total trip duration of LTs (1/x transformed) decreased significantly with chick age (LMM, estimate ± SE: −0.003 ± 0.001) and differed significantly between years. It was also affected significantly by a random effect (bird identity) (Table 2). *Post-hoc* estimated marginal means test revealed that LTs in 2011 were significantly longer in duration (mean ± SD: 21.4 ± 4.8 h; N = 5) compared to 2016 (mean ± SD: 16.3 ± 6.5 h, N = 29; p = 0.025) and 2018 (mean ± SD: 16.1 ± 7.7 h, N = 9; p = 0.016). LTs duration in 2016 and 2018 were similar (p = 0.558).

### Characteristics of foraging locations

**Figure 4:**
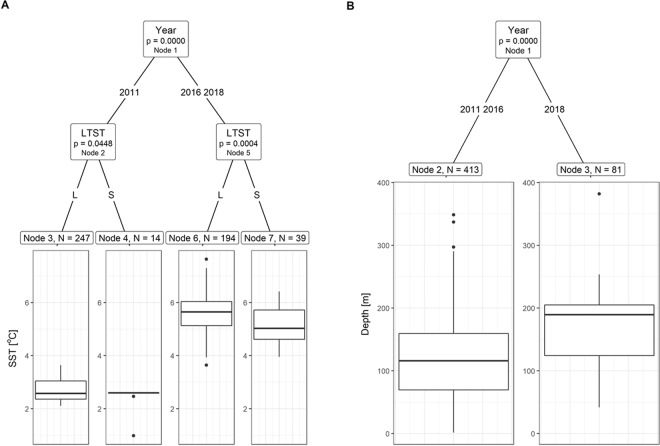
A Conditional Inference Tree characterizing factors affecting the sea surface temperature (SST) (**A**) and sea depth (**B**) in foraging locations of GPS-logger-equipped chick-rearing little auks breeding in Hornsund in 2011, 2016 and 2018. The following factorial predictors were used as initial predictors: year (2011, 2016, 2018), and foraging trip type (LTST with L – long trips and S - short trip). Encircled variables have the strongest association to the response variable. The p values listed at each encircled node represent the test of independence between the listed variable and the response variable [SST (**A**) or sea depth (**B**)]. Terminal nodes indicate variable levels characterizing the response variable and N indicates the number of foraging locations corresponding to specific predictors levels. Boxplots show the median (band inside the box), the first (25%) and third (75%) quartile (box), the lowest and the highest values within 1.5 interquartile range (whiskers) and outliers (circles). Plot was created in R software version 3.5.2^77^.

CIT revealed that SST at foraging locations differed in regard to year and trip type (Fig. 4A). All foraging locations in 2011 were characterized by lower temperatures compared to 2016 and 2018. Then, foraging locations were further split in regard to the trip type (Node 2 and 5), showing that mean SST values were significantly higher at LTs compared to STs locations (by 0.24 °C in 2011, and by 0.48 °C in 2016 and 2018) (Fig. 4A).

CIT also revealed that sea depth at foraging locations differed in regard to year but not to the trip type (Fig. 4B). Foraging locations in 2018 were situated in deeper sea areas compared to 2011 and 2016 (Fig. 4B).

**Figure 5:**
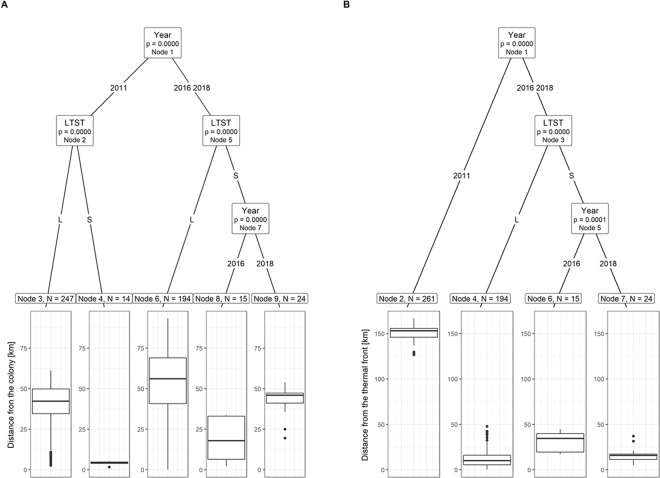
A Conditional Inference Tree characterizing factors affecting the following characteristics of the foraging locations of GPS-logger-equipped chick-rearing little auks breeding in Hornsund in 2011, 2016 and 2018: distance to the colony (**A**) and distance to the nearest thermal front (**B**). The following factorial predictors were used as initial predictors: year (2011, 2016, 2018), and foraging trip type (LTST with L – long trip and S - short trip). Encircled variables have the strongest association to the response variable. The p values listed at each encircled node represent the test of independence between the listed variable and the response variable [distance to the colony (**A**) or the nearest thermal front (**B**)]. Terminal nodes indicate variable levels characterizing the response variable and N indicates the number of foraging locations corresponding to specific predictors levels. Boxplots show the median (band inside the box), the first (25%) and third (75%) quartile (box), the lowest and the highest values within 1.5 interquartile range (whiskers) and outliers (circles). Plot was created in R software version 3.5.2^77^.

Applying CIT analysis to examine the distance of foraging locations from the colony in regard to year and trip type (Fig. 5A), we found that both year and trip type were significant factors. In 2011 (Node 2) foraging locations visited during STs were located significantly closer to the colony compared to those visited during LTs. In 2016 and 2018 (Node 5), although overall birds foraged further from the colony, also during STs forged significantly closer to the colony compared to LTs. Then, STs latter locations differed significantly between the years (Node 7) with those visited in 2016 being located closer to the colony compared to 2018 (Fig. 5A).

Finally, CIT revealed that the distance of foraging locations from thermal fronts was different in regard to year and trip type (Fig. 5B). In 2011 foraging locations were located significantly further from the thermal fronts compared to other two years. In 2016 and 2018 (Node 3) birds during LTs foraged closer to the thermal fronts compared to STs. Then, locations at STs differed significantly between the years (Node 5) with those in 2018 located closer to the thermal fronts compared to 2016 (Fig. 5B).

### Similarity between main feeding areas of GPS-tracked birds

We found a higher main feeding areas (the 95% utilisation densities) overlap of trips of GPS-tracked little auks performed in 2016 and 2018 [Bhattacharyya’s affinities (BA) = 0.68 compared to those performed in 2011 and 2016 (BA = 0.58) and 2011 and 2018 (BA = 0.51).

**Figure 6:**
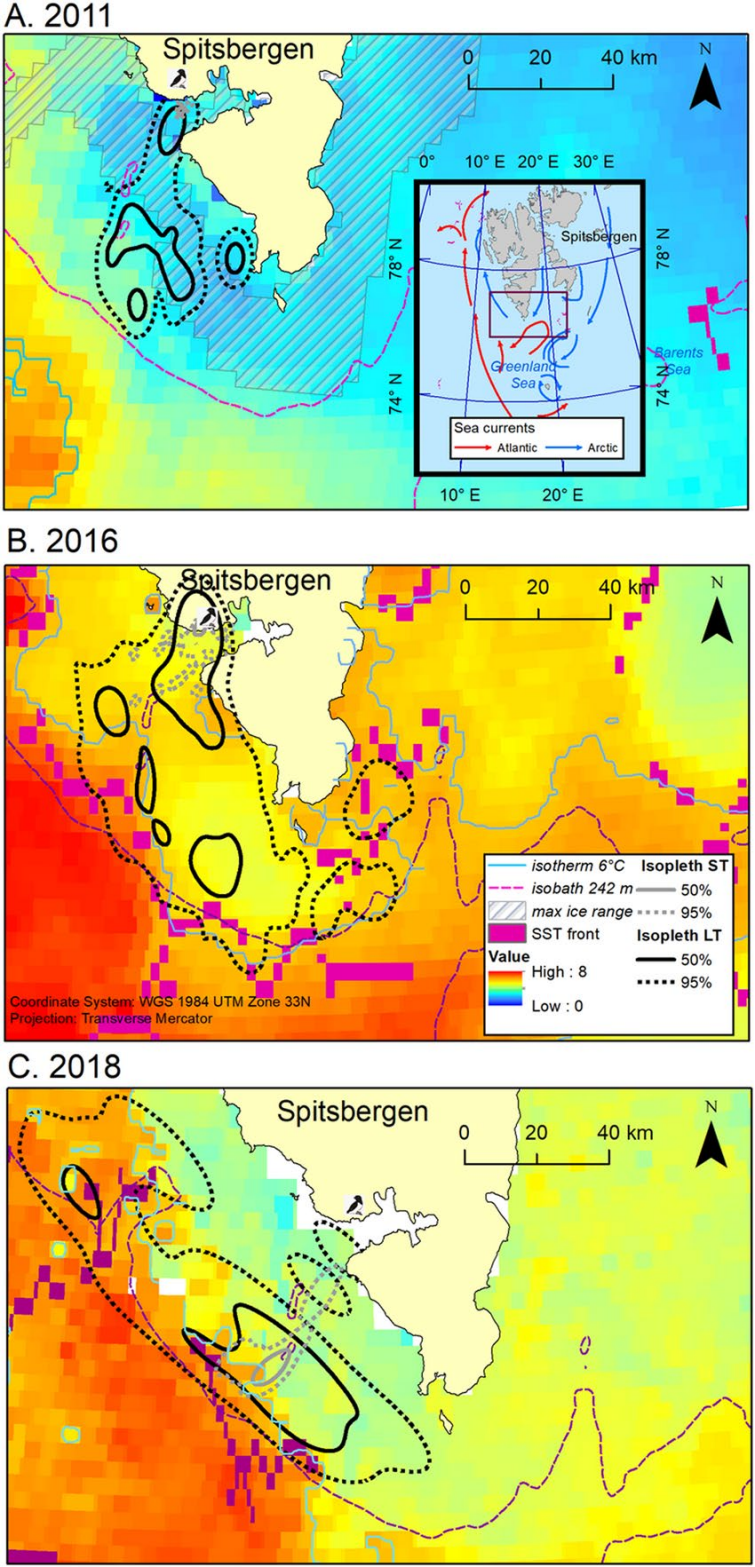
Utilization densities (UD) (95% for main feeding areas and 50% for core breeding areas) of chick-rearing GPS-equipped little auks breeding in Hornsund in 2011 (**A**) 2016 (**B**), and 2018 (**C**). Sea surface temperature (SST) maps – monthly mosaic for July based on MODIS Aqua satellite data^53^]; mosaic for July 2016 reconstructed based on mosaics for July and August 2016 (see details in the text). Thermal fronts detected in SST monthly mosaics for July using Cayula & Cornillon algorithm^64,65^ with a 1 °C threshold in sea surface temperature (SST). A 242 m isobath reflects the multiyear cut-off point value for Arctic zooplankton occurrence in the Hornsund area^20^. Isotherm 6 °C (for the studied period in particular years) reflects physiological threshold for *Calanus glacialis* functioning in Svalbard^39^. Isopleth ST and LT – 50% and 95% utilisation density isopleths for short and long trips, respectively. Maps were produced in ArcMap 10.3.1 (Redlands, CA: Environmental Systems Research Institute). Isobath created based on, global relief model of Earth’s surface IBCAO ver. 3^54^. Source of sea ice extent: Multisensor Analyzed Sea Ice Extent - Northern Hemisphere (MASIE-NH), Version 1^55^.

Comparison of main feeding areas of LTs and ST revealed the highest similarity in 2018 (BA = 0.55), intermediate in 2016 (BA = 0.36) and lowest in 2011 (BA = 0.10) (Fig. 6).

### Chick diet composition

Arctic copepod, *Calanus glacialis* CV was the most important prey item in all studied years, constituting 73–88% of the total abundance, and 68–90% of total energy value of food loads delivered for the chicks (Table 3). Its average abundance and energy content per one food load differed significantly among the studied years with the highest values recorded in 2018 compared to 2011 and 2016. It is average abundance and energy content were similar in 2016 and 2016 (Fig S1 and S2). The second important prey item was the other developmental stage of *Calanus glacialis* – adult females, constituting 6–10% of the total energy in 2011 and 2016 (Table 3), but much less (0.1) in 2018 (Fig. S1). The *Calanus finmarchicus* CV, copepod associated with Atlantic water masses, constituted considerable part of energetic value of food loads only in 2016 (when it made up 4.6% of the total energy content; 1.5% in 2011 and 2% in 2018); we found significant differences in average energy content of *Calanus finmarchicus* CV among all studied years (Fig. S1). Then, amphipod *Themisto abyssorum* was recorded in all years but constituted considerable part of energetic value of food loads in 2016 and 2018 (when it made up 1.8% and 1.6% of the total energy content); however its average energy content differed significantly between 2011 (0.2%) and 2018 (Fig. S1). Sea ice-associated amphipod *Apherusa glacialis* was recorded exclusively in the coldest 2011, present in 85.2% food samples collected from adults and constituting 7.6% of total energy content (Table 3, Fig. S1 and S2). Average abundance and energy content per food load for other important prey items (euphausiids *Thysanoessa inermis*, *Thysanoessa longicaudata*) was similar in the studied years (Fig. S1 and S2).

**Table 3:** Relative abundance and energy content [%] of the most important (≥1.5% of total energy content) prey items in food loads collected from adult little auks in the second week of chicks life. Prey items ranked by relative abundance/energy content in all years combined.

Prey item	Abundance [%]			Energy content [%]		
	2011	2016	2018	2011	2016	2018
*C. glacialis CV*	**83.6**	**72.8**	**88.2**	**67.6**	**75.8**	**89.7**
*C. glacialis AF*	**5.6**	2.6	0.1	**9.5**	**5.6**	0.2
*Thysanoessa inermis*	0.2	0.1	<0.1	**9.0**	**6.7**	0.1
*Apherusa glacialis*	1.5	—	—	**7.6**	—	—
*C. finmarchicus CV*	**5.3**	**12.9**	**5.6**	1.5	4.6	2.0
*Themisto abyssorum*	0.1	0.8	0.7	0.2	1.8	1.6
*Thysanoessa longicaudata*	<0.1	<0.1	0.2	<0.1	0.5	1.8
Other prey items	3.6	10.8	5.2	4.6	4.9	4.6
N samples	27	20	20	27	20	20

Prey items constituting >5% of total abundance or energy content are bolded.

Total energy value of the whole food load ranged from 313 to 2815 kJ g dw^−1^ (median = 1151, IQR: 316, n = 67 samples) and did not differ significantly among the studied years (Kruskal-Wallis test, p = 0.096).

### Chick survival and growth parameters

In the group of control nests with successfully hatched chicks, chick survival up to 20 d was similar in all the years: 2011 (96%, n = 89); 2016 (92%, n = 92); 2018 (89%, n = 64; Fisher’s exact test with Monte Carlo simulation, p = 0.335).

However, peak body mass (Box-Cox transformed), when controlled for the peak body mass day (LM, F = 0.02, p = 0.879), differed significantly between the studied years (F = 10.51, p < 0.001). *Post-hoc* Tukey test with Holm correction revealed that peak body mass was the highest in 2011 when compared with 2018 (p < 0.001) and 2016 (p = 0.0496), while comparing it between 2016 and 2018 the difference was not significant (p = 0.214; Table 4).

**Table 4:** Mean ± SD peak body mass and fledging day of the little auk chicks from the colony in Hornsund in 2011, 2016 and 2018.

Year	Mean	SD	Min	Max	Mean	SD	Min	Max	N
Peak body mass [g]					Peak body mass day				
2011	130	9.1	99	148	19	2.4	13	26	66
2016	124	9.2	101	138	20	2.7	16	25	18
2018	119	13.3	91	143	20	3.7	14	28	28
**Fledging mass [g]**					**Fledging day**				
2011	118	10.0	95	139	24	1.8	20	28	64
2016	112	7.4	103	129	27	1.5	25	29	11
2018	114	11.3	90	143	25	2.0	21	28	23

Fledging mass, when controlled for fledging day (that was its significant predictor being lower in chicks longer staying in the nest, estimate ± SD: −1.92 ± 0.53; F = 13.05, p = 0.0005), tended to be affected by the year (F = 2.68, P = 0.074), with the highest value in recorded in 2011 (Table 4).

Chick’s age at the moment of achieving peak mass (19–20 days) was similar in the all the studied years (GLM, p = 0.290), and not related to peak mass (p = 0.203; Table 4). Similarly, chicks age at fledging (24–27 days) was similar in all the studied years (GLM, p = 0.410) and not related to fledging mass (p = 0.975; Table 4).

## Discussion

Our multidisciplinary work, combining GPS-tracking, remote sensing, breeding biology and diet composition, features the first comprehensive investigation of foraging trips of GPS-tracked little auks breeding in the same colony in years with contrasted oceanographic conditions. We found that despite considerable inter-annual differences in environmental conditions little auks breeding in Hornsund performed foraging flights of quite similar characteristics. Chicks were also provided with food of similar energy value and had similar survival rate in all studied years. However, we did find some differences in foraging flight performance and the chicks’ peak and fledging body mass, suggesting that the birds’ flexibility is somehow restricted.

### Characteristics of little auks foraging trips and feeding areas

Maximal range of all foraging flights (median 39.9 km, IQR: 50.8, N = 81 flights) observed in our study is similar to distance recorded in the same colony during the incubation period [median 55.4 km IQR: 50.4, N = 11 flights^80^; Mann-Whitney *U* test, *U* = 311, p = 0.107]. However, it is lower than in the Magdalenefjorden colony [median 129 km^38^] characterized by generally higher water temperatures in close foraging grounds. This suggests that the Hornsund area serve as relatively stable and favourable environment for the breeding little auks.

Our study indicates that foraging trip duration of little auks breeding in Hornsund, being positively related to maximal flight range and total distance covered, is a function of the foraging location. This is an important support for the so-far rarely directly tested hypothesis that long foraging trips of the little auk are related to exploration of more distant foraging grounds. Previous studies based on data from the little auk equipped with time-depth recorders (TDR) has also reported distant foraging trips for Hornsund (130 km^42^) but due to nature of the applied devices, the distance was only estimated, based on the trip duration.

The average maximal flight range reported here for chick rearing little auks is surprisingly high, given expected high cost of flight for the species^81,82^. However, data from GPS-tracked individuals that lost their chicks (two recorded flights) were even more distant: 123–129 km from the colony (Fig. 1B). One failed breeder from Bjørnøya performed even further foraging trip towards cold water zone situated 299 km from the colony^50^. All these results together demonstrate that little auks are able to cover occasionally a long distance to favourable foraging grounds. Apparently however, very long flights cannot be performed on a regular basis due to considering constraints of regular chick feedings. Studies on other seabirds also indicate that failed breeders often continue to associate with the colony, operating as central-place foragers but expand their foraging areas^83^.

**Figure 7:**
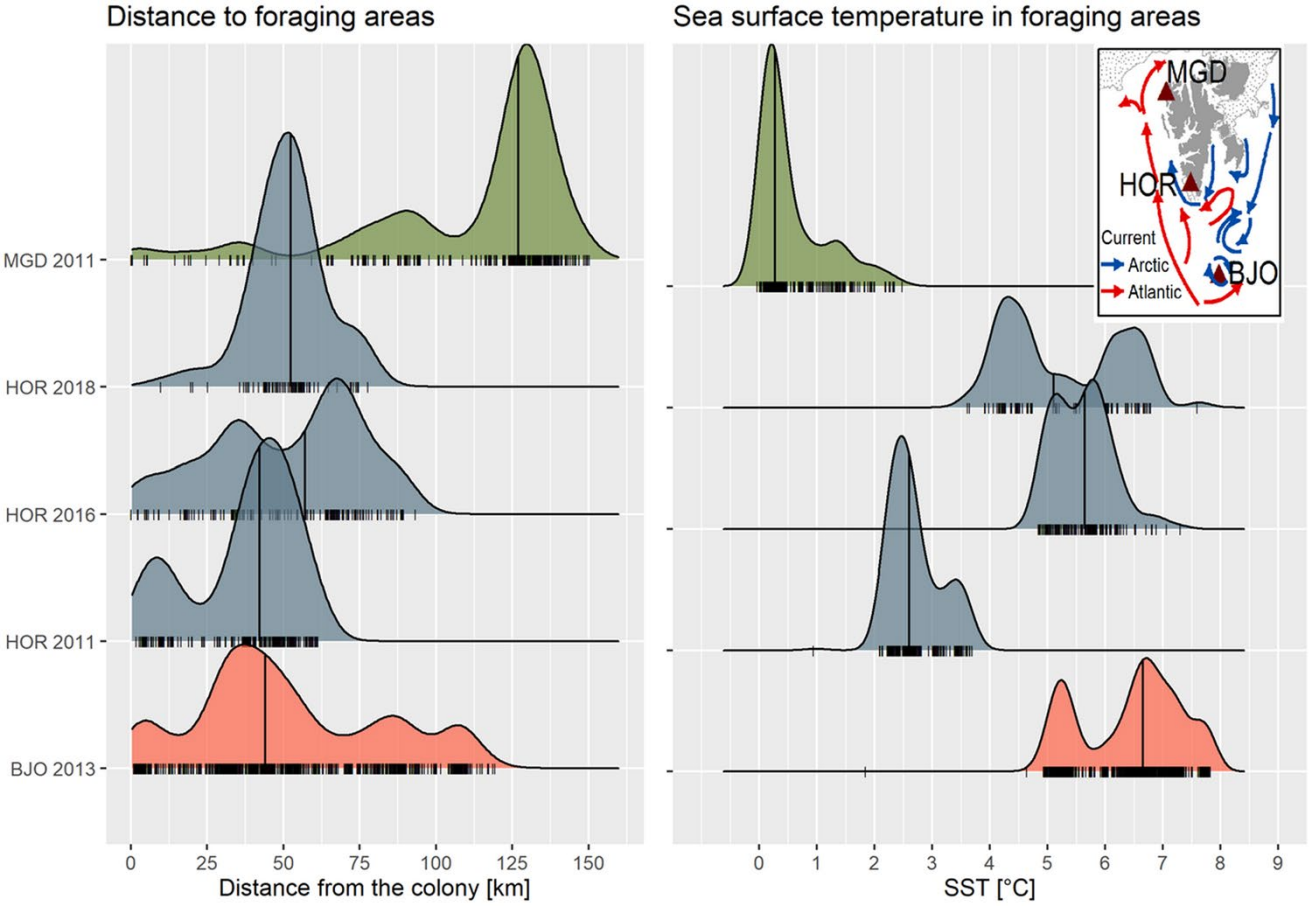
Probability densities for distances from the colony to foraging positions and sea surface temperatures (SST) in the foraging areas of GPS-tracked little auks during the chick-rearing period in three colonies in Svalbard (colony location, sea ice extent in 2011 and main sea currents shown in the embeded map); Codes: BJO 2013 - Bjørnøya in 2013^50^, MGD 2011 – Magdalenefjorden in 2011^38^, HOR – Hornsund in 2011^38^, 2016 and 2018 (this study). Horizontal lines represent median values, rugs plots represent distributions of data in particular sites and years. Plot was created in R software version 3.5.2^77^.

Our study revealed considerable inter-year differences in SST in the vicinity of the local little auk colony but despite these differences birds foraged mostly in cold water masses. (median 3.5 °C). This is understandable given the fact that SST < 5.1 °C has been recognized as a range optimal for the Arctic zooplankton community occurring in the Hornsund area^39^; its abundance greatly depletes when SST increases^49,67^. However, little auks also happened to forage in warm waters − 19% and 39.5% of foraging locations in 2016 in 2018, respectively were recorded in water with SST > 6 °C warm areas. Also in other Svalbard colonies (Bjørnøya, Isfjorden, Kongsfjorden and Magdalenefjorden) the birds were reported to forage in relatively high SST values (4–7 °C; Fig. 7) and still to deliver mainly *C. glacialis*, despite conditions being optimal for the boreal counterpart, *C. finmarchicus*^25,27,50,84,85^. This apparent puzzle can be explained by foraging in frontal zone or underwater eddies. Those oceanographic features often offer attractive feeding conditions and indeed are exploited regularly by seabirds, including little auks^38,86^.

Majority of the foraging positions were recorded within the depth range preferred by Arctic zooplankton communities in the Hornsund area (up to bottom depth −242 m^39^). However, some (0.8–7.2%) foraging positions were located in deep water zone (Fig. 2). This suggests that birds might have foraged on the open sea prey, amphipod *Themisto abyssorum* recorded mainly in 2016 and 2018. This, in turn, suggests that birds could have adjusts their foraging behaviour to the environmental conditions – when abundance of preferred food items was lower in the proximity of the colony, birds foraged at different, more distant, and deeper locations.

Our results suggest the importance of some oceanographic features as the foraging grounds for little auks. The location of core feeding areas of LTs and the majority of foraging locations correspond to Arctic-origin cold water masses over the shelf zone rich in Arctic-type zooplankton community with large copepods^39,56^. Studied little auks foraged in warm years also in the thermal front zone at shelf break area (often characterized by elevated stocks of zooplankton^87^). Observed distribution of foraging areas is consistent with results from at-sea surveys performed during the chick-rearing period^17,88,89^ in the Hornsund shelf area. The little auks in the coldest 2011 foraged also in the marginal ice zone (MIZ). This habitat offers energy-rich prey, i.e. *C. glacialis* and the sympagic amphipod *Apherusa glacialis*^49,90,91^. The latter prey species has been recorded in diet samples from 2011 and other seasons with presence of MIZ within the range of cost-effective flights from the Hornsund colony^92,93^. Since MIZ was present within the main feeding area explored in all seasons, foraging in this temporal habitat did not alter the maximal range of foraging flights in 2011. Some foraging locations (i.e. 6.9% in 2011, 3.9% in 2016) were situated in distance ≤ 5 km from the colony, i.e. in the inner part of the Hornsund fjord what is in concordance with observations during boat surveys^94^. Central and inner parts of the fjord also offer Arctic zooplankton, however, less abundant compared to its outer part and inner glacial bays^95^.

The 8.0 h cut-off value of LT and ST duration derived in this study corresponds well to 7.6–10.0 h calculated for two colonies in NW Spitsbergen^42,44,96^. The mean durations of STs (3.8 h) and LTs (16.8 h) found in this study are longer than in previous seasons in Hornsund (ST: 1.3–2.0 h and LT; 11.7–13.0 h), or in E Greenland (ST: 1.9–2.0 h and LT: 9.6–9.8 h)^42,45^ but comparable with data from Bjørnøya (ST: 3.9 h and LT: 22.7 h^50^) and NW Spitsbergen (Magdalenefjorden: ST: 3.3 h and LT: 14.7 h;^44^ Kongsfjorden: ST: 2.4–2.5 h and LT: 17.0–19.8 h^42,45^) characterized by higher SST. Again, all this variety in duration of little auks foraging trips demonstrate their considerable flexibility.

We did not found any significant inter-annual differences in distance or duration of STs, either distance of LTs. This unexpected result may be explained by efficient finding pockets of cold water in their traditional foraging areas, even in suboptimal feeding conditions^26^ what has been reflected in our study by high contribution of the preferred food, *C. glacialis*, in all years. Surprisingly, we found significant inter-annual differences in LT duration with the longest values in the coldest 2011. Considering closer location of optimal cold-water foraging grounds to the colony and high chick peak body mass in this year we would interpret these longer LTs as an extended resting period of adults related to their self-feeding.

Chick diet was dominated by high energy prey item, *Calanus glacialis* CV and this is concordant with the previous studies from the same and other colonies in western Spitsbergen^18,19,27,56,97^. Diet in the cold 2011 was characterized by presence of ice-associated amphipod *Apherusa glacialis*, previously detected in the little auk diet samples from Hornsund only once, in a 2004 with MIZ presence in the vicinity of the colony^92^. Surprisingly, we found the highest abundance of *C. glacialis* CV in the warmest 2018. As already partly discussed above, it may be explained by little auks ability to effectively forage in the high SST areas like frontal zone or deeper water cold core eddy, rich in favourable prey^26,86^. Besides, the contribution of *C. glacialis* CV was the lowest in 2011, because the diet was supplemented by other energy rich prey as *Calanus glacialis* AF, *Apherusa glacialis* or *Thysanoessa inermis*, likely to be abundant in the close vicinity of the colony due to quite exceptional ice conditions.

### Consequences of foraging conditions for little auk reproductive performance

Little auks buffered suboptimal conditions in the foraging areas, by foraging in various oceanographic features and so provisioning with food loads of the similar energetic value as in more favourable conditions. However, lower peak body mass and tendency for lower fledging mass in warmer years indicate that birds could have a trouble to successfully complete the breeding and some breeding parameters and/or adults survival could have been compromised. An experimental study on little auks with increased flight costs via feather clipping revealed that the parental birds have some limited ability to adjust their reproductive effort in a given breeding attempt; chicks with a clipped parent had lower peak and fledging mass^40^. All those results suggest that despite flexible foraging, provisioning rates in suboptimal conditions are not fully maintained. The lowest peak body mass recorded in 2018 may be also explained by the unusual high rainfall during the chick rearing period [142 mm of the total rainfall in July vs 40 mm for the multi-year mean for 1979–2017 (Meteorological Bulletin of Polish Polar Station in Hornsund)]. A negative impact of precipitation on the growth of chicks younger than 24 d has been found in the same colony^61^. A negative relationship between precipitation and fat reserves in 9–13 day old chicks^98^ and positive with level of corticosterone^99^ may be explained by excessive heat loss of the chicks due to their down becoming sodden^61^.

### Little auks foraging in the future

Lack of considerable inter-annual differences in majority of foraging flight parameters and chicks survival may be explained by the fact that observed variability of environmental and trophic conditions was still in the range of little auk foraging behaviour flexibility.

Despite episodes with low water temperature and sea ice events in summer observed in some recent years in the Hornsund area^24,30,93^, the long-term hydrographic monitoring shows that along the west coast of Spitsbergen, SST and volume of Atlantic waters, though variable, are steadily increasing^29^. Foraging flexibility observed in little auks breeding in Svalbard (Fig. 7) and results of recent modelling of future foraging habitats in this region^37^ suggest that those planktivorous birds may be temporarily resilient to moderate climate changes of the Arctic marine environment. They may start feeding on novel zooplankton species or even small fish currently extending their distributions in the North Atlantic. Nevertheless, predicted long-term changes [including even a sea-ice-free Arctic scenario towards the end of 21^st^ century^14^] will result in considerable deterioration of the foraging habitats of little auks with inevitable negative consequences for this species and whole Arctic marine and terrestrial ecosystems^37^. Ongoing “Atlantification”, of the European Arctic^100^ resulting in changes in zooplankton communities structure^101,102^ has already affected some avian top predators on Svalbard^100^.

### Limitations of our study

We are aware of possible limitations of our study. Firstly, a part of our study based on behaviour of GPS-tracked individuals. The behaviour and energetics of such individuals may be affected by externally attached devices by expending extra energy countering both the additional mass and the increased drag, and by decreasing some aspects of flight performance^103,104^. Little auks with the same type of GPS-logger as in the present study performed longer duration foraging trips^105^, or more frequent LTs^96^ compared to unburdened individuals. This bias might have also affected the pattern of foraging trips observed in our study. However, as we used the same type of loggers in all seasons, one may expect the same potential bias in all years. Since we found that in our study body mass of chicks in experimental nests with birds equipped with GPS-loggers was lower compared to control nests (Supplementary materials Fig. S3), we compared chick growth only in the control group of nests.

Secondly, due to heavy cloudiness it was impossible to generate maps of SST for July 2018 and we reconstructed monthly composite for this month based on small area with recorded values. Those reconstruction may not be fully representative for real conditions met by GPS-tracked little auks. However, in this part of the Barents Sea the water masses follow the bottom topography stabilizing the position of the Polar Front on the shelf break^35^. Thus, our reconstructed SST mosaic should represent the general spatial pattern of environmental conditions during earlier phases of the little auks’ chick-rearing period.

## Conclusions

Little auks breeding on SW Spitsbergen exploited various microhabitats (marginal ice zone, cold water zone, thermal front zones, warm waters), allowing them to secure their chicks with energy-rich food despite considerable inter-annual variability of environmental conditions in the foraging areas. It corroborates previous studies indicating that they are able to respond to a wide range of environmental conditions and prey availability by exploring various oceanographic features^18,24,26,37,38,50,97,105,106^. Chick survival was high in all studied seasons. However, in the warmer years chick peak body mass was lower compared to the cold year suggesting that provisioning rates may not have been fully maintained. Buffering the suboptimal foraging conditions by searching for preferred Arctic prey among very abundant but energetically suboptimal boreal counterparts in warmer water microhabitats may have long-term negative consequences for little auks (lower adults survival). Thus, ongoing “Atlantification” of the European Arctic means in a longer perspective inevitable negative consequences for little auks inducing serious negative implications for the whole marine and terrestrial Arctic ecosystems.

## Supplementary information

Supplementary files.

## Data Availability

The datasets generated during and/or analysed during the current study are available from the corresponding author upon reasonable request.
