# Supplementary material for: Flexibility of little auks foraging in various oceanographic features in a changing Arctic
Source: Sci Rep. 2020 May 19;10:8283. doi: 10.1038/s41598-020-65210-x (PMC7237489; doi:10.1038/s41598-020-65210-x)
Supplement: Supplementary file 1 — Supplementary files. [file 41598_2020_65210_MOESM1_ESM.docx]

**Supplementary Materials**

**Flexibility of little auks foraging in various oceanographic features in a changing Arctic** Dariusz Jakubas^1, *^, Katarzyna Wojczulanis-Jakubas^1^, Lech M. Iliszko^1^, Dorota Kidawa^1^, Rafał Boehnke^2^, Katarzyna Błachowiak-Samołyk^2^, Lech Stempniewicz^1^

^1^ Department of Vertebrate Ecology and Zoology, Faculty of Biology, University of Gdańsk, Wita Stwosza 59, PL-80-308 Gdańsk, Poland

^2^ Institute of Oceanology, Polish Academy of Sciences, Powstańców Warszawy 55, PL-81-712 Sopot, Poland

**GPS deployment**

Table S1. Number of deployed GPS loggers, survival of chicks in nests with instrumented parents

| Parameter | 2011 | 2016 | 2018 | Total |
| --- | --- | --- | --- | --- |
| No. loggers deployed | 13 | 14 | 10 | 37 |
| No. loggers used more than once after charging | 0 | 1 | 2 | 3 |
| No. individuals with deployed logger (A) | 13 | 15 | 12 | 40 |
| No. ind. with collected data (B) | 10 | 15 | 12 | 37 |
| % ind. with collected data (B/A) | 76.9 | 100.0 | 100.0 | 92.5 |
| No. nests with inds with deployed loggers (C) | 13 | 12 | 11 | 36 |
| No. nest with two parents sequentially instrumented (D) | 0 | 3 | 1 | 4 |
| % nest with two parents sequentially instrumented (D/C) | 0 | 25.0 | 9.1 | 11.1 |
| No. nests with inds with collected data (E) | 10 | 12 | 11 | 33 |
| % nests with inds with collected data (E/C) | 76.9 | 100.0 | 100.0 | 91.7 |
| No. inds with records too short for analyses (F) | 5 | 2 | 3 | 10 |
| % inds with records too short for analyses (F/B) | 50.0 | 13.3 | 25.0 | 27.0 |
| Total no. birds with analysed records (G) | 7 | 12 | 9 | 28 |
| % of all inds deployed (G/A) | 53.8 | 80.0 | 75.0 | 70.0 |
| % of inds with collected data (G/B) | 70.0 | 80.0 | 75.0 | 75.7 |
| Total no. of trips recorded (H) | 7 | 64 | 23 | 94 |
| No. trips excluded (I) | 0 | 13 | 0 | 13 |
| % of trips analysed (H-I)/H | 100.0 | 79.7 | 100.0 | 86.2 |
| No. trips excluded (I) | 7 | 13 | 23 | 43 |
| % of trips analysed (H-I)/H | 0.0 | 79.7 | 0.0 | 54.3 |
| No. of inds with retrieved loggers (J) | 3 | 2 | 6 | 11 |
| % inds with lost logger (J/A) | 23.1 | 13.3 | 50.0 | 27.5 |
| No. inds with lost logger (K) | 2 | 1 | 2 | 5 |
| % inds with lost logger (K/A) | 15.4 | 6.7 | 16.7 | 12.5 |
| No. alive chicks during 2 controls after deployment (L) | 12 | 9 | 11 | 32 |
| % of alive chicks (L/C) | 92.3 | 75.0 | 100.0 | 88.9 |
| No. dead chicks (M) | 1 | 2 | 0 | 3 |
| No. of dead chicks in nests of inds that collected data | 0 | 2 | 0 | 2 |
| % of dead chicks (M/C) | 7.7 | 16.7 | 0.0 | 8.3 |
| No. chicks of unknown status (N) | 0 | 1 | 0 | 1 |
| % chicks of unknown status (N/C) | 0.0 | 8.3 | 0.0 | 2.8 |

**Diet composition**


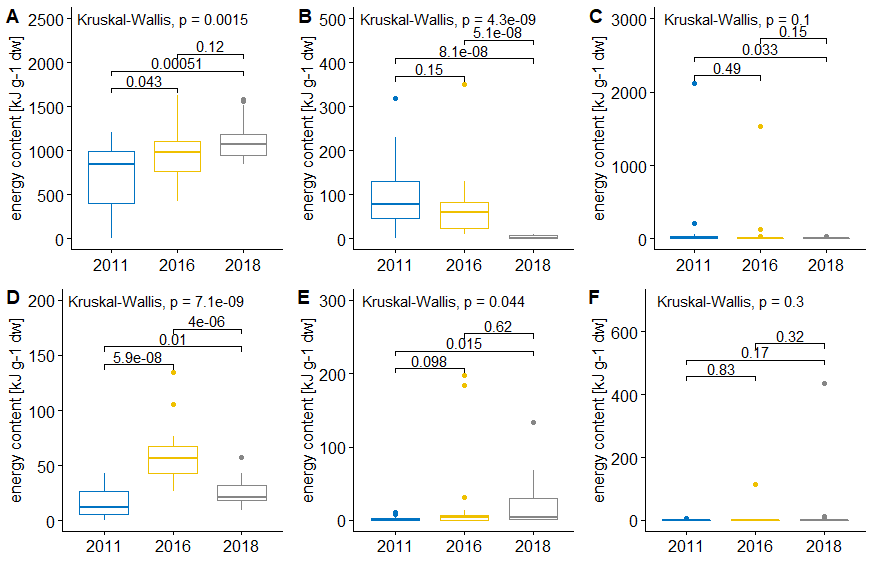


Fig. S1. Energy content of the most important (≥1.5 % of total energy content) prey items (A. *Calanus glacialis CV*, B. *Calanus glacialis* AF, C. *Thysanoessa inermis*, D. *Calanus finmarchicus* CV, E. *Themisto abyssorum*, F. *Thysanoessa longicaudata*) in the little auks food loads. Lines indicate results of Mann-Whitney U test. Boxplots show the median (band inside the box), the first (25%) and third (75%) quartile (box), the lowest and the highest values within 1.5 interquartile range (whiskers) and outliers (circles). Plot was created in R software version 3.5.2 ^1^.


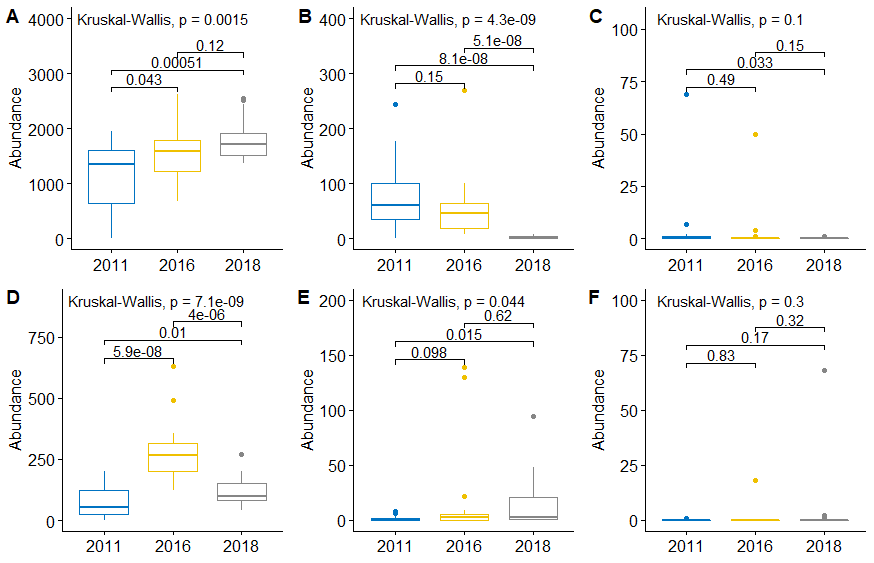
 Fig. S2. Abundance (number of items per food load) of the most important (≥1.5% of total energy content) prey items (A. *Calanus glacialis CV*, B. *Calanus glacialis* AF, C. *Thysanoessa inermis*, D. *Calanus finmarchicus* CV, E. *Themisto abyssorum*, F. *Thysanoessa longicaudata*) in the little auks food loads. Lines indicate results of Mann-Whitney U test. Boxplots show the median (band inside the box), the first (25%) and third (75%) quartile (box), the lowest and the highest values within 1.5 interquartile range (whiskers) and outliers (circles). Plot was created in R software version 3.5.2 ^1^.

**Effect of GPS loggers on the chicks growth rate**

Here we compared chick growth in experimental (with the parent deployed with GPS logger) and control nests in two years 2016 and 2018. Year 2011 was excluded from this analysis because chick measurements routine was considerably shorter compared to the other two seasons. However, chick body mass variables (peak body mass, fledging mass, and body mass recession) ln 2011 were similar in nests of instrumented and control parents; Chick survival in experimental nest (78%) was lower, but not significantly, than in control nests (96%) (Appendix 1 in ^2^).

We analysed chicks body mass initially considering it in the following model (LMM, with ML function; *lmer* function from the package l*merTest*, ^3^): chick body mass ~ chick age category + year + nest type (experimental vs control) + year * nest type + (1|Nest). There were four chick age categories, established based on chicks day of life (6-9, 10-13, 14-15 and 16-19 days of life). For parents GPS status there were two categories: control (no GPS on any parents), and experimental (at least one parent deployed with the logger; we did not differentiate the nests were two parents were burden with logger or a parent was re-deployed with the logger due to a small sample size for these cases). Due to multiple measurements of the same chicks during the season, we included chick’s identity (nest) in the model.

To test the effect of chicks identity we compared the model with and without the random factor we performed Kenward-Roger approximation using *pbkrtest* R package ^4^. Results of this analysis (Table S2) showed that indeed that factor was important so it was retained in the final model. Since neither season or its interaction with parents GPS status were significant in the initial model, we excluded them, and run the analysis with the rest of the parameters.

Thus, in the final model we considered as explanatory variables: chick age category, parents GPS status and (1|Nest) and only chick age and chick identity were significant (Table S3).

To test the difference between control and experimental chicks in particular age categories we performed a bootstrap procedure with 1000 iterations (separately for each age category), and considered the difference significant if it was <= 0 in <0.05 cases. Results of these bootstrap testing revealed that significant difference between control and experimental nests were found only for the chicks at age of 6-9 (P = 0.023) and 17-19 days of life (P = 0.0) (Fig. S3).

Table S2. Linear mixed effects models estimating the effects of year (factorial: 2011, 2016, 2018), nest type (factorial: experimental vs control), chick age (continuous) and bird identity (random effect) on characteristics of foraging flights of GPS-tracked little auks. Significance of random effect, chick identity (r.e. chick identity) estimated by *F*-test with Kenward-Roger approximation. Significant effects are bolded.

| Parameter | NumDF | DenDF | F | P |
| --- | --- | --- | --- | --- |
| Chick Age | 3 | 177.78 | 370.66 | **<0.001** |
| Year | 1 | 230.33 | 0.03 | 0.869 |
| Nest type | 1 | 59.90 | 3.67 | 0.060 |
| Year x nest type | 1 | 30.50 | 0.27 | 0.602 |
| r.e. chick identity* | 6 | 192.76 | 183.90 | **<0.001** |

* r.e. – random effect - estimated by Kenward-Roger approximation

Table S3 Linear mixed effects models estimating the effects of nest type (factorial: experimental vs control), chick age (continuous) and bird identity (random effect) on characteristics of foraging flights of GPS-tracked little auks. Significance of random effect, chick identity (r.e. chick identity) estimated by *F*-test with Kenward-Roger approximation. Significant effects are bolded.

| Parameter | NumDF | DenDF | F | P |
| --- | --- | --- | --- | --- |
| Chick Age | 3 | 175.45 | 366.76 | **<0.001** |
| Nest type | 1 | 57.93 | 3.38 | 0.071 |
| r.e. chick identity* | 4 | 171.88 | 278.53 | **<0.001** |

* r.e. – random effect, estimated by Kenward-Roger approximation


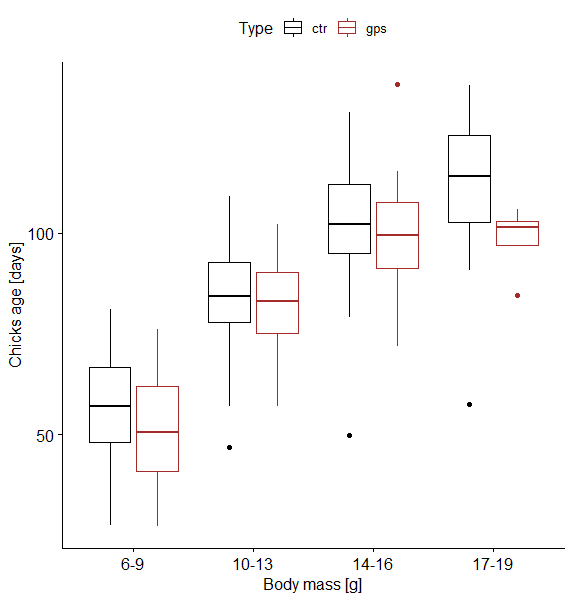


Fig. S3. Body mass of little auk chicks in experimental (gps – nest with individuals equipped with GPS logger) and control (ctr) nests. Boxplots show the median (band inside the box), the first (25%) and third (75%) quartile (box), the lowest and the highest values within 1.5 interquartile range (whiskers) and outliers (circles). Plot was created in R software version 3.5.2 ^1^.

**References**

1. R Core Team. R: A Language and Environment for Statistical Computing. R Foundation for Statistical Computing, Vienna, Austria. (2018).

2. Jakubas, D. *et al.* Foraging closer to the colony leads to faster growth in little auks. *Mar. Ecol. Prog. Ser.* **489**, 263–278 (2013).

3. Kuznetsova, A., Brockhoff, P. B. & Christensen, R. H. B. lmerTest Package: Tests in Linear Mixed Effects Models. *J. Stat. Softw.* **82**, 1–26 (2017).

4. Halekoh, U. & Højsgaard, S. A Kenward-Roger Approximation and Parametric Bootstrap Methods for Tests in Linear Mixed Models - The R Package pbkrtest. *J. Stat. Softw.* **59**, (2014).
